# Supplementary material for: Iron Deficiency and Restless Sleep/Wake Behaviors in Neurodevelopmental Disorders and Mental Health Conditions
Source: Nutrients. 2024 Sep 11;16(18):3064. doi: 10.3390/nu16183064 (PMC11435348; doi:10.3390/nu16183064)
Supplement: Supplementary file 1 [file nutrients-16-03064-s001.zip › nutrients-3117862-supplementary.pdf]

nutrients

MDPI

Table S1. Questions and response options for intake forms provided to patients/caregivers. All content on the intake forms was approved by the Provincial Health Services Authority.

**1 - Consent**	*Consent form outlining type of information collected and how collected information will be used - all questions were approved by the Provincial Health Services Authority*<br><br>"I have read and understand this form. I voluntarily consent to PHSA collecting, using and disclosing the information I provide."<br>+<br>"Would you like to be contacted about future opportunities to participate in research studies at the BCCH Sleep Clinic? (your response will not affect your current care)"	"I consent (proceed to intake form in survey format)" OR "I do not consent (please exit from survey"<br>+<br>"Yes" OR "No"
**2 - Introduction**	Ethnic Background	Black, East/Southeast Asian, Vietnamese, Indigenous, Middle Eastern, South Asian, White, Do not know, Another race category, Prefer not to answer
**3 – Description of Sleep and Wake Behaviors**	Child's current/working diagnoses	Open-ended
	How long has your child's sleep been a concern?	<3 months, >3 months, >1 year, Since birth, N/A
	Select the main areas of concern related to your child's sleep	Bedtime, Excessive daytime behaviors, Awakenings, Routines, Sleep disordered breathing, N/A
	How would you rate your child's sleep quality?	Excellent, Good, Average, Poor, Very poor, N/A
	What is the current well-being/quality of life of your child?	Slider scale (excited, happy, neutral, sad, devastated)
	How would the child's well-being improve if your child's sleep problems improved/resolved?	Slider scale (excited, happy, neutral, sad, devastated)
	What is your level of distress as the caregiver?	Slider scale (not distressed – very distressed)
	If your child's sleep problems improved/resolved, what would your level of distress be as a caregiver?	Slider scale (not distressed – very distressed)
	Do you as a caregiver have sleep problems?	Yes, No, N/A

	What are the three main goals that yourself (caregiver) and/or the patient would like to achieve?	Open-ended w/ slider scale (0-10 level of importance)
	What are the three main concerns that yourself (caregiver) and/or the patient has?	Open-ended w/ slider scale (0-10 level of importance)
	Do you have any additional goals or concerns for yourself or the patient?	Open-ended
	Does your child currently take any medications?	Yes (please list), No, N/A
	Is there any additional information that you want to disclose? You can write as much or as little as you want! If you have nothing to add, you can write N/A	Open-ended
**4 – Nighttime and Sleep Behaviors (Questionnaire)**	SDSC Questionnaire +<br>Your child's mood at bedtime +<br>Your child's mood after a night time awakening +<br>Your child's mood when they wake up for the day +<br>If you selected "I don't know" for any of the questions, please specify +<br>Was there anything related to your responses that you want to mention? You can write as much or as little as you want! If you have nothing to add, you can write "N/A"	Domains covered: DIMS, SDB, Arousal disorders, SWTD, Disorders of excessive somnolence, Sleep hyperhidrosis
**5 – Daytime Behaviors (Questionnaire)**	ADHD Rating Scale IV +<br>If you selected "Not Applicable" for any of the questions, please specify +<br>Is there any additional information that you would like to disclose? You can write as much or as little as you want! If you have nothing to add, you can write "N/A"	Subscales of Inattention and Hyperactivity-impulsivity

Table S2. Results from a multivariate logistic regression analysis analyzing the relationship between RLS with ID (with/without a family history of ID), ADHD, ASD, bedtime resistance, restlessness before falling asleep, restlessness during sleep, age, and sex.

Outcome: RLS				
**Effect**	**Odds Ratio**	**95% Confidence Interval**		**P-Value**
**Iron Deficiency**<br>Family history ID vs no ID	6.25	1.46	26.74	0.013
**Iron Deficiency**<br>No family history of ID vs no ID	2.67	0.68	10.39	0.158
**ASD**<br>Yes vs no	0.88	0.41	1.86	0.728
**ADHD**<br>Yes vs no	2.12	1.01	4.42	0.046
**Bedtime Resistance**<br>Per point on scale	1.17	0.91	1.51	0.224
**Restlessness Before Falling Asleep**<br>Per point on scale	1.09	0.84	1.42	0.508
**Restlessness During Sleep**<br>Per point on scale	1.08	0.86	1.36	0.502
**Age**<br>Per year older	0.98	0.91	1.05	0.556
**Sex**<br>Male vs Female	0.75	0.36	1.55	0.436

Table S3. Multivariate logistic regression analysis examining the relationship between familial RLS with ID (with/without a family history of ID), ADHD, ASD, bedtime resistance, restlessness before falling asleep, restlessness during sleep, age, and sex.

**Outcome: Familial RLS**				
**Effect**	**Odds Ratio**	**95% Confidence Interval**		**P-Value**
**Iron Deficiency**<br>Family history of ID vs no ID	4.38	1.01	18.94	0.048
**Iron Deficiency**<br>No family history of ID vs no ID	1.51	0.36	6.38	0.573
**ASD**<br>Yes vs no	1.71	0.90	3.24	0.103
**ADHD**<br>Yes vs no	1.14	0.62	2.12	0.675
**Bedtime Resistance**<br>Per point on scale	1.13	0.91	1.41	0.257
**Restlessness Before Falling Asleep**<br>Per point on scale	1.02	0.82	1.27	0.882
**Restlessness During Sleep**<br>Per point on scale	1.06	0.87	1.29	0.591
**Age**<br>Per year older	0.97	0.91	1.03	0.314
**Sex**<br>Male vs Female	0.65	0.35	1.22	0.181

Table S4. Multivariate logistic regression analysis examining the relationship between painful RLS with ID (with/without a family history of ID), ADHD, ASD, bedtime resistance, restlessness before falling asleep, restlessness during sleep, age, and sex.

**Outcome: Painful RLS**				
**Effect**	**Odds Ratio**	**95% Confidence Interval**		**P-Value**
**Iron Deficiency**<br>Family history of ID vs no family history of ID	2.20	0.83	5.82	0.113
**ASD**<br>Yes vs no	1.38	0.53	3.61	0.515
**ADHD**<br>Yes vs No	1.70	0.65	4.42	0.279
**Bedtime Resistance**<br>Per point on scale	0.80	0.57	1.14	0.222
**Restlessness Before Falling Asleep**<br>Per point on scale	0.91	0.66	1.25	0.549
**Restlessness During Sleep**<br>Per point on scale	0.98	0.73	1.31	0.890
**Age**<br>Per year older	0.96	0.86	1.07	0.480
**Sex**<br>Male vs Female	0.89	0.35	2.27	0.804

Table S5. Odds ratios of having a family history of ID & (ADHD or ASD) with various sleep/wake-disorders.

	ADHD	ASD
**Insomnia/DIMS**	3.4444<br>(1.3728 to 8.6425)<br>p = 0.0084	4.7727<br>(1.8245 to 12.4847)<br>p = 0.0014
**EDS/DOES**	1.8000<br>(0.7344 to 4.4120)<br>p = 0.1988	1.9847<br>(0.7537 to 5.2264)<br>p = 0.1653
**CRSD**	0.6000<br>(0.1874 to 1.9206)<br>p = 0.3895	0.6187<br>(0.2153 to 1.7781)<br>p = 0.3727
**Parasomnias**	0.8710<br>(0.3295 to 2.3022)<br>p = 0.7806	0.9236<br>(0.3037 to 2.8085)<br>p = 0.8886
**SDB**	1.2222<br>(0.5347 to 2.7939)<br>p = 0.6343	0.5492<br>(0.2364 to 1.2758)<br>p = 0.1635
**Non-Restorative Sleep**	1.0800<br>(0.4734 to 2.4637)<br>p = 0.8549	0.7257<br>(0.3149 to 1.6723)<br>p = 0.4516
**SWTD**	4.4118<br>(0.8400 to 23.1699)<br>p = 0.0794	5.9211<br>(0.6626 to 52.9093)<br>p = 0.1115
**PLMS/Restlessness**	1.8095<br>(0.7500 to 4.3657)<br>p = 0.1869	1.6790<br>(0.6806 to 4.1423)<br>p = 0.2607
**RLS**	7.0000<br>(1.4882 to 32.9259)<br>p = 0.0138	4.0533<br>(1.3325 to 12.3296)<br>p = 0.0137
**Probable Painful RLS**	3.4839<br>(0.9868 to 12.2997)<br>p = 0.0525	1.8743<br>(0.5617 to 6.2539)<br>p = 0.3068
**Familial RLS**	5.9825<br>(2.3547 to 15.1990)<br>p = 0.0002	2.8182<br>(1.1661 to 6.8111)<br>p = 0.0214
**ASD**	2.0057<br>(0.8595 to 4.6805)<br>p = 0.1074	n/a
**Genetic Conditions**	0.3077<br>(0.0330 to 2.8664)	0.4179<br>(0.1007 to 1.7333)

	p = 0.3006	p = 0.2293
**Externalizing Disorders**	n/a	1.1875<br>(0.5063 to 2.7854)<br>p = 0.6928
**ADHD**	n/a	1.2847<br>(0.5531 to 2.9842)<br>p = 0.5601
**Internalizing Disorders**	1.5918<br>(0.6816 to 3.7176)<br>p = 0.2827	0.9660<br>(0.4195 to 2.2244)<br>p = 0.9352
**Intellectual Disability**	1.5918<br>(0.2361 to 2.6147)<br>p = 0.6942	0.6476<br>(0.2355 to 1.7807)<br>p = 0.3999
**Neurologic Conditions**	0.7412<br>(0.2446 to 2.2457)<br>p = 0.5964	0.5509<br>(0.1941 to 1.5640)<br>p = 0.2628
**SPD**	0.3022<br>(0.0782 to 1.1678)<br>p = 0.0827	0.6250<br>(0.1874 to 2.0843)<br>p = 0.4444
**SIB**	1.3429<br>(0.3607 to 4.9997)<br>p = 0.6603	1.0968<br>(0.4299 to 2.7981)<br>p = 0.8467
**Iron Deficiency**	n/a	n/a
**Family History of Iron Deficiency**	n/a	n/a
**RLS with PLMs (based on clinical description of restlessness)**	n/a	5.8333<br>(1.5416 to 22.0737)<br>p = 0.0094

Table S6a. Number of patients taking various medications across different categories: all patients, patients without ID, patients with ID, patients without a family history of ID, and patients with a family history of ID. .

	All patients N=199	Patients without ID N=11	All patients with ID N=188		
			All patients with ID N=188	No family history of ID N=107	Family history of ID N=81
**No medications**	**32 (16.1%)**	**2 (18.2%)**	**30 (16.0%)**	15 (14.0%)	15 (18.5%)
**Sleep (including melatonin)**	**69 (34.7%)**	**4 (36.4%)**	**65 (34.6%)**	34 (31.8%)	31 (38.3%)
**Melatonin only**	**67 (33.7%)**	**3 (27.3%)**	**37 (19.7%)**	19 (17.8%)	18 (22.2%)
**Antihistamines**	**6 (3.0%)**	**0 (0%)**	**6 (3.2%)**	4 (3.7%)	2 (2.5%)
**Non-stimulants**	**62 (31.2%)**	**1 (9.1%)**	**61 (32.4%)**	35 (32.7)	26 (32.1%)
**Stimulants**	**52 (26.1%)**	**4 (36.4%)**	**48 (25.5%)**	25 (23.4%)	23 (28.4%)
**Antipsychotics**	**42 (21.1%)**	**2 (18.2%)**	**40 (21.3%)**	24 (22.4%)	16 (19.8%)
**Antidepressants (includingTrazodone)**	**79 (39.7%)**	**5 (45.5%)**	**74 (39.4%)**	45 (42.1%)	29 (35.8%)
**Benzodiazepines**	**11 (5.5%)**	**2 (18.2%)**	**9 (4.8%)**	4 (3.7%)	5 (6.2%)
**All antiseizure medications**	**32 (16.1%)**	**1 (9.1%)**	**31 (16.5%)**	19 (17.8%)	12 (14.8%)
**Gabapentin/Carbamazepine/ Pregabalin**	**27 (13.6%)**	**0 (0%)**	**27 (14.4%)**	17 (15.9%)	10 (12.3%)
**Diabetes/thyroid/growth hormone**	**7 (3.5%)**	**0 (0%)**	**7 (3.7%)**	5 (4.7%)	2 (2.5%)
**Iron (alone or in combination with other medications)**	**27 (13.6%)**	**4 (36.4%)**	**23 (12.2%)**	14 (13.1%)	9 (11.1%)
**Iron & proton pump inhibitors**	**6 (3.0%)**	**1 (9.1%)**	**5 (2.7%)**	3 (2.8%)	2 (2.5%)
**Others/ Antihistamines/ Iron/ proton pump inhibitors- all others have been excluded from this group**	**15 (7.5%)**	**0 (0%)**	**15 (8.0%)**	10 (9.3%)	5 (6.2%)

Table S6b. Number of patients taking melatonin in combination with various medications across different categories: all patients, patients without ID, patients with ID, patients without a family history of ID, and patients with a family history of ID. .

	**All patients N=199**	**Patients without ID N=11**	**All patients with ID N=188**		
			**All patients with ID N=188**	No family history of ID N=107	Family history of ID N=81
**No melatonin**	**132 (66.3%)**	**8 (72.7%)**	**124 (66.0%)**	74 (69.2%)	50 (61.7%)
**Melatonin & sleep**	**67 (33.7%)**	**3 (27.3%)**	**64 (34.0%)**	33 (30.8%)	31 (38.3%)
**Melatonin & antihistamines**	**2 (1.0%)**	**0 (0%)**	**2 (1.1%)**	1 (0.9%)	1 (1.2%)
**Melatonin & non-stimulants**	**28 (14.1%)**	**0 (0%)**	**28 (14.9%)**	15 (14.0%)	13 (16.0%)
**Melatonin & stimulants**	**21 (10.6%)**	**2 (18.2%)**	**19 (10.1%)**	10 (9.3%)	9 (11.1%)
**Melatonin & antipsychotics**	**17 (8.5%)**	**0 (0%)**	**17 (9.0%)**	9 (8.4%)	8 (9.9%)
**Melatonin & antidepressants**	**34 (17.1%)**	**2 (18.2%)**	**32 (17.0%)**	20 (18.7%)	12 (14.8%)
**Melatonin & benzodiazepines**	**2 (1.0%)**	**0 (0%)**	**2 (1.1%)**	1 (0.9%)	1 (1.2%)
**Melatonin & antiseizure**	**9 (4.5%)**	**0 (0%)**	**9 (4.8%)**	3 (2.8%)	6 (7.4%)
**Melatonin & lithium/ anti inflammatory/ antiviral therapy**	**28 (14.1%)**	**2 (18.2%)**	**26 (13.8%)**	16 (15.0%)	10 (12.3%)
**Melatonin & diabetes/thyroid/growth hormone**	**2 (1.0%)**	**0 (0%)**	**2 (1.1%)**	1 (0.9%)	1 (1.2%)
**Melatonin & iron**	**9 (4.5%)**	**2 (18.2%)**	**7 (3.7%)**	3 (2.8%)	4 (4.9%)
**Iron (alone or in combination with other medications)**	**27 (13.6%)**	**4 (36.4%)**	**23 (12.2%)**	14 (13.1%)	9 (11.1%)

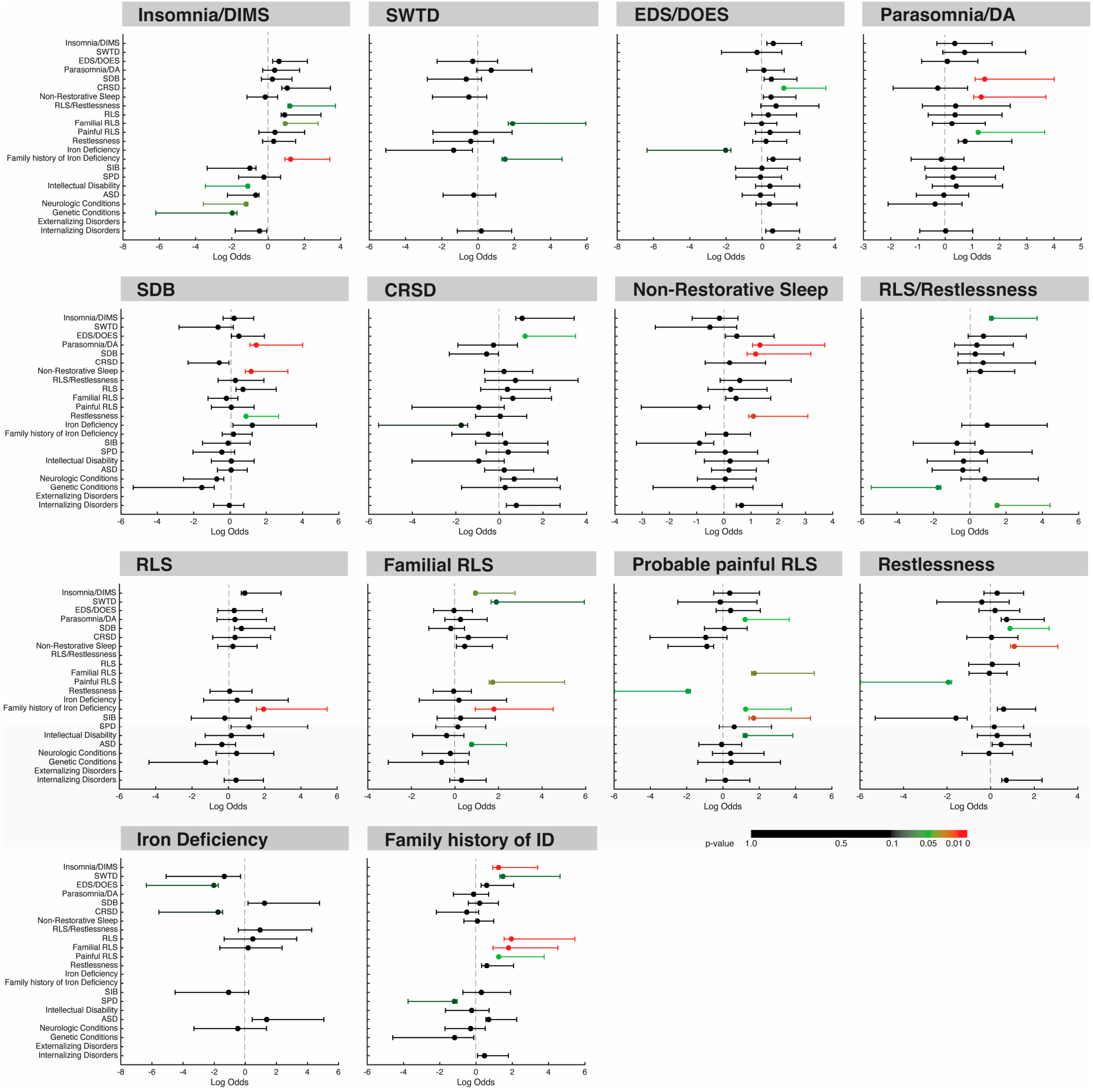

Figure S1. Visual representation of the log(odds ratio) in the subgroup analysis of ADHD. Error bars represent the 95% confidence intervals. Heat maps representing *p*-values were used to optimize readability. CRSD: circadian rhythm sleep disorders; DA: disorders of arousal; EDS/DOES: excessive daytime sleepiness/disorders of excessive somnolence; RLS: restless legs syndrome; SDB: sleep disordered breathing; SIB: self-injurious behaviours; SPD: sensory processing disorder; SWTD: sleep wake transition disorders.

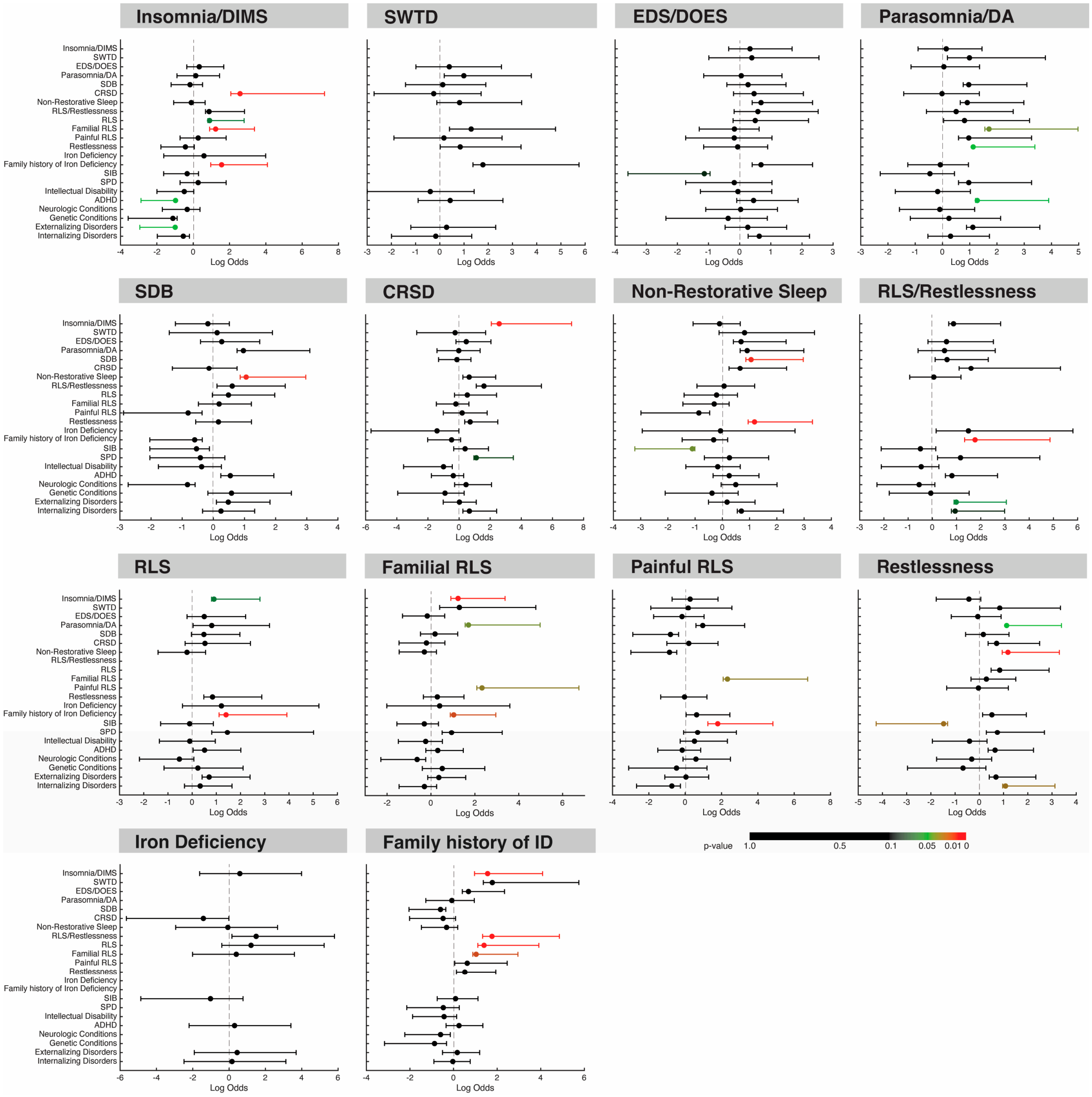

Figure S2. Visual representation of the log(odds ratio) in the subgroup analysis of ASD. Error bars represent the 95% confidence intervals. Heat maps representing p-values were used to optimize readability. CRSD: circadian rhythm sleep disorders; DA: disorders of arousal; EDS/DOES: excessive daytime sleepiness/disorders of excessive somnolence; RLS: restless legs syndrome; SDB: sleep disordered breathing; SIB: self-injurious behaviours; SPD: sensory processing disorder; SWTD: sleep wake transition disorders.

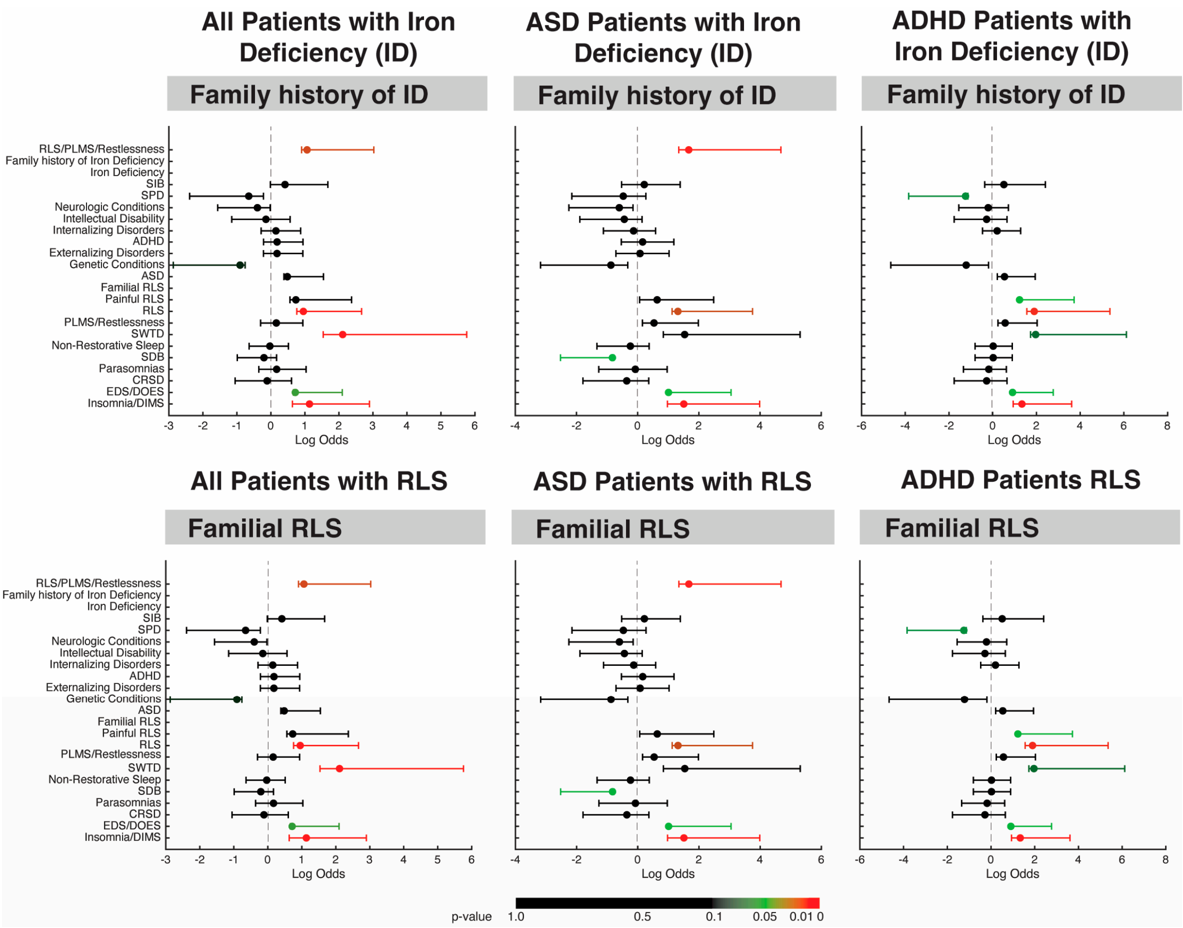

Figure S3. Visual representation of the log(odds ratio) based on family history of ID and familial RLS. Error bars represent the 95% confidence intervals. Heat maps representing *p*-values were used to optimize readability. CRSD: circadian rhythm sleep disorders; DA: disorders of arousal; EDS/DOES: excessive daytime sleepiness/disorders of excessive somnolence; RLS: restless legs syndrome; SDB: sleep disordered breathing; SIB: self-injurious behaviours; SPD: sensory processing disorder; SWTD: sleep wake transition disorders.
